# Supplementary material for: When months matter; modelling the impact of the COVID-19 pandemic on the diagnostic pathway of Motor Neurone Disease (MND)
Source: PLoS One. 2023 Jan 27;18(1):e0259487. doi: 10.1371/journal.pone.0259487 (PMC9882907; doi:10.1371/journal.pone.0259487)
Supplement: Attachment — Submitted filename: NewResponse.docx [file pone.0259487.s001.docx]

We would like to thank the reviewers for their helpful comments the response are below:

1. The formatting and referencing are now consistent with PLOS One guidelines.
2. The funding information is now updated and in the right place on the form.
3. Our data availability statement has been amended accordingly.
4. The corresponding author is affiliated with the chosen institute, Nikhil Sharma at UCL.
5. The titles and authors are now identical on both forms.

Specifically reviewer 1:

**Reviewer #1: This very interesting paper explores the impact of the pandemic on the MND diagnostic
pathway, suggesting that the COVID-19 pandemic will result in later-stage diagnoses and more
emergency presentations of undiagnosed MND.
I would propose only minor changes:**

1. Introduction section: The statement that “There are nearly 1100 people diagnosed with MND each
year [...]” appear to ben not clear. I would specify in which region.

Done

2. Implications for secondary healthcare providers section: I would add in this section a small paragraph
discussing the available opportunity to manage ALS patients remotely through telemedicine tools
(suggested refences: Bombaci A, et al. Telemedicine for management of patients with amyotrophic
lateral sclerosis through COVID-19 tail. Neurol Sci. 2021; Vasta R, et al. (2020) Telemedicine for patients
with amyotrophic lateral sclerosis during COVID-19 pandemic: an Italian ALS referral center experience.
Amyotrophic Lateral Sclerosis and Frontotemporal Degeneration)

This is an important point and we have added this to the discussion.

Reviewer #2: In this manuscript, Burchill et al., considering that the COVID-19 pandemic has caused
significant delays in NHS pathways, especially for caring disabling diseases needing multidisciplinary
approaches, such as amyotrophic lateral sclerosis (ALS), and the majority of GP appointments occurred
online with subsequent delays in secondary care assessment, applied a model to describe the pre-
COVID-19 diagnostic pathway and then introduced plausible COVID-19 delays by using Monte Carlo
simulation accounting for a wide range of delays. The pre-COVID model reproduced known features of
the MND diagnostic pathway, with a median time to diagnosis of 399 days and predicting 5.2% of MND
patients present as undiagnosed emergencies. COVID-19 resulted in diagnostic delays from 558 days
when only primary care was 25% delayed, to 915 days when both primary and secondary care were
75%. The model predicted an increase in emergency presentations ranging from 15.4%-44.5%. The
model suggests the COVID-19 pandemic will result in later-stage diagnoses and more emergency
presentations of undiagnosed MND.
This is an interesting paper, focused on an innovative and crucial topic for clinical practice in the MND
field. It is well written and well discussed. I have only a minor suggestion.

Among implications for secondary healthcare providers, the use of home-monitoring and services at
home, such as through technological devices aimed at improving the assistance and quality of life of the
monitored patients (i.e. telemonitoring respiratory function and telehealth services, such as TiM system
by Hobson et al., 2018 DOI: 10.1080/21678421.2018.1440408), could be discussed in this regard,
although considering the heterogeneity in healthcare services in UK, as well as in other countries

Similar to review 1, we have added this to the discussion.
